# Running demands and tactical individual actions of wingers appear to depend on the playing formations within an amateur football team

**DOI:** 10.1038/s41598-023-36157-6

**Published:** 2023-06-01

**Authors:** José María Izquierdo, Diego Marqués-Jiménez, Juan Carlos Redondo

**Affiliations:** 1 Valoración del Rendimiento Deportivo, Actividad Física y Salud y Lesiones Deportivas (REDAFLED), Department of Didactics of Musical, Plastic and Corporal Expression, Faculty of Education, University of Valladolid, Soria, Spain; 2 Department of Physical Education and Sports, University of León, León, Spain

**Keywords:** Environmental social sciences, Energy science and technology

## Abstract

We examine the differences in running performance demands and tactical individual actions for male amateur football wingers in four tactical formations: 4-2-3-1, 4-4-2, 3-5-2 and 3-4-2-1 during an entire season. Running demands were assessed in terms of: total distance run; distance traveled at 3 different speed ranges (Jogging, Running, and Sprinting); and number of accelerations registered in two different magnitude bands (Medium and High) while tactical individual actions were assessed through 3269 team match observations. 3-5-2 formation entails the highest running demands, exhibiting significant disparities in Jogging when compared to 4-2-3-1 and 3-5-2, in Sprinting when comparing 4-2-3-1 with both the 3-5-2 and the 3-4-2-1 formations. Moreover, the wingers in the 3-5-2 formation demonstrate the lowest scores in various tactical individual actions, including Shots: 3-5-2 vs. 3-4-2-1; Goal Area Shots: 3-5-2 vs. 3-4-2-1; and Dribbles: 3-5-2 vs. 3-4-2-1. Finally, wingers registered the highest levels of defensive tactical individual actions in 4-4-2 and 3-5-2. Findings suggest it would be of benefit for coaches to focus on formulating specific training plans to address the specific demands placed on wingers playing in these amateur matches and running performance demands and tactical individual actions should be considered together with tactical formation.

## Introduction

Knowledge of the physical and physiological demands placed on football players during matches is key to the development of effective training programs^1^. In recent years technological advances have enabled ever more detailed profiling of players’ skills-related and physical performance so facilitating the analysis of the physical, tactical, and technical demands on players^2^. One particular technology, the global positioning system (GPS), has proved particularly important providing data that can be used to measure, monitor and assess the various external loads experienced by players^3^.

Identifying the parameters that truly impact match outcomes remains one of the key concerns in modern football^4^. In this line, the most commonly studied external load measures are power output, distance, speed, acceleration/deceleration, time-motion analysis, and strength function^5^. The analysis of match running performance has been conducted for various purposes, including to distinguish the profiles of different playing positions, or to assess the acute fatigue, but it has been argued that more tactical-strategic perspectives need to be considered^6^ due to the nature of the game. Investigations that relate physical demands and tactical individual actions during football matches are very limited. In this line, the technical-tactical element of football means that the physical demands on players are multifactorial^7^ and vary depending on an individual player’s role and associated on-pitch tasks^8^ or the team’s playing formation^9,10^.

During professional football matches, elite players can cover distances of around 10,000–11,000 m^11^. However, significant differences in this figure exist depending on playing position with wide and central midfielders recording the greatest total running distances: 11,900 m and 12,027 m respectively^12^. Findings from other work shows that compared to other playing positions, full-backs and wingers always register highest for high-intensity distance runs and the number of high-intensity runs or sprints regardless of tactical formation^13,14^. Concerning tactical systems, Tierney et al.^15^ demonstrated differences in player performance demands between five playing formations (4-4-2, 4-3-3, 4-2-3-1, 3-5-2 and 3-4-3), with the 3-5-2 requiring the highest physical demands as measured by several metrics including the total distance run and number of activities involving high intensity activity, and high metabolic load.

With respect to players’ tactical individual actions, Bradley et al.^16^ showed, in an elite context, that the largest values for the total number of passes made are achieved by central defenders and midfielders, as compared with full-backs, wingers, and strikers. Other works show that while player profiles remain largely unchanged, the tactical system employed in a given game can affect the overall number of passes observed with higher volumes of passes seen for 4-4-2 formation compared to either 4-3-3 or 4-5-1^9^. Due to most of physical efforts finishing in the wide zones of the opponent field^17^ it is very likely that wingers have a bigger impact in the goalscoring zones because they perform in an opened position no related with build up and organizing play but perform more assistant passes and dribbles than the players in other positions.

What is lacking from current research is an investigation of the relationship between running performance demands and tactical individual actions during games^9^. This is an important consideration since physical factors may impact players’ technical performance and engagement in tactical actions and vice-versa. Moreover, there is still a lack of research into the influence of tactical playing formation on physical performance and tactical individual actions undertaken, and this is particularly the case in the amateur context. This article attempts to address these gaps in the literature and hence it examines the game play of an amateur football team over an entire season to determine whether or not team formation affects running performance demands and tactical individual actions. More specifically, this work focusses on the performance of wingers and compares four team formations: 4-2-3-1, 4-4-2, 3-5-2 and 3-4-2-1. On the basis of the previous studies mentioned above in professional context, we can hypothesize that wingers in 3-5-2 will require the highest physical demands, and in 4-4-2 and 3-4-2-1 will perform high actions related to the goal: shots on target and dribbles.

## Methods

### Study design

The data for this study was obtained from a total of 34 official matches (the complete 2021–2022 season) played by a team in an amateur male football senior league. Over the season, the team used four tactical systems: 4-2-3-1, 4-4-2, 3-5-2 and 3-4-2-1 (obviously, without including goalkeepers in these descriptions). GPS technology and a tailored observation methodology were used to collect data concerning wingers’ running performance and tactical individual actions, respectively. A combination of a video system and a high frequency GPS presents a good potential for the task analysis in football^18^. Descriptive statistics were used to compare player running performance and tactical individual actions for each of the different tactical formations. This study was conducted in accordance with the Declaration of Helsinki and obtained the ethical approval of the Ethics Committee of the of the University of León (ULE-022–2021). All players and coaches were notified of the research procedures, requirements, and benefits before giving their written informed consent.

### Sample

**Figure 1:**
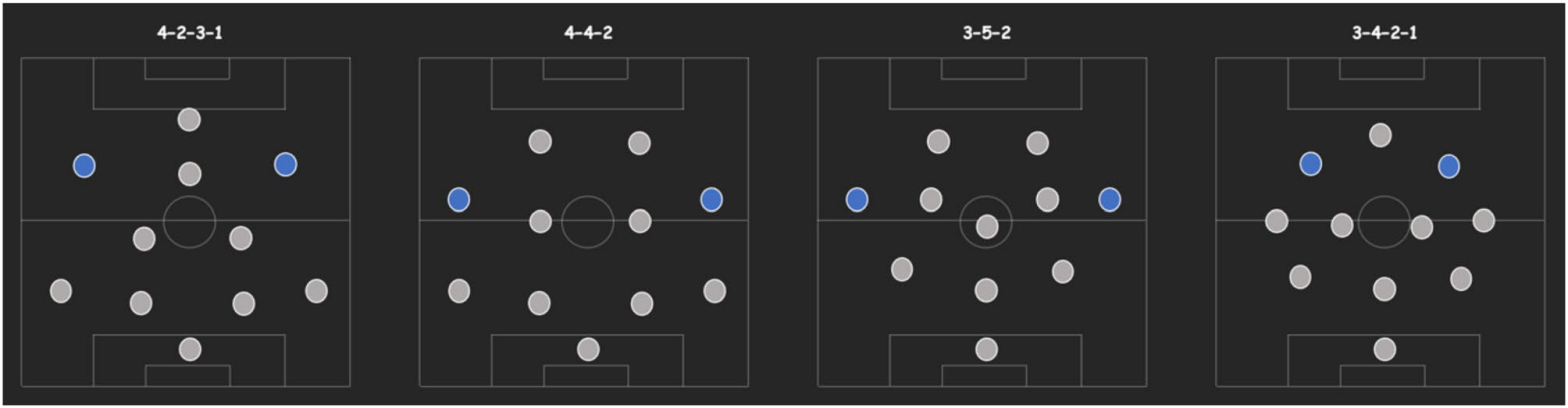
Winger playing positions in each tactical system: indicated by the blue dots.

Participants included all players who played as wingers during the season of interest giving a total of 7 individual players. The average age of participants was 25.3 ± 4.67 years and their average body mass and height were 77.2 ± 7.12 kg and 1.76 ± 0.16 respectively (where all values are expressed as a mean ± standard deviation (SD). Over the season there were a total of 68 instances of game-play (34 games with 2 wingers per game). Figure 1 shows the wingers’ playing positions (blue dots) in each of the tactical systems used by the study-team over the playing season.

The total playing time for the season studied was 3202 min during which 3269 team match-observations of tactical individual actions were recorded.

### Running performance demands

Players’ movements were recorded using a minimax S4 GPS tool (Catapult Innovations, Melbourne, Australia) sampling at 10 Hz and a compatible triaxial accelerometer. This equipment was worn in a small vest on the upper back of each player. Typical error data for these units suggest a coefficient of variation (CV) across a range of speeds of 3.1–8.3% at a constant velocity, and 3.6–5.9% when accelerating^19^. This system has been used in other work to record data such as total distances run by players and the distances run at high intensity^20^.

Six variables were studied here: the total distance (kilometers) run (TD); the distance (meters) covered at three different speed ranges: Jogging, between 14.4 and 19.8 kmh^−1^ (D > 14.4); Running, between 19.9 and 25 kmh^−1^ (D > 19.8), and Sprinting, above 25.0 kmh^−1^ (D > 25); and numbers of accelerations recorded in the range 2–4 ms^−2^ (Medium accelerations, Acc2-4) and above 4 ms^−2^ (High accelerations, Acc > 4) as used previously by Arjol-Serrano et al.^9^. Data was downloaded post-match using the Apex V 1.2 software package and exported to Excel (V.16.54. Microsoft Corporation) for analysis.

### Tactical individual actions

**Figure 2:**
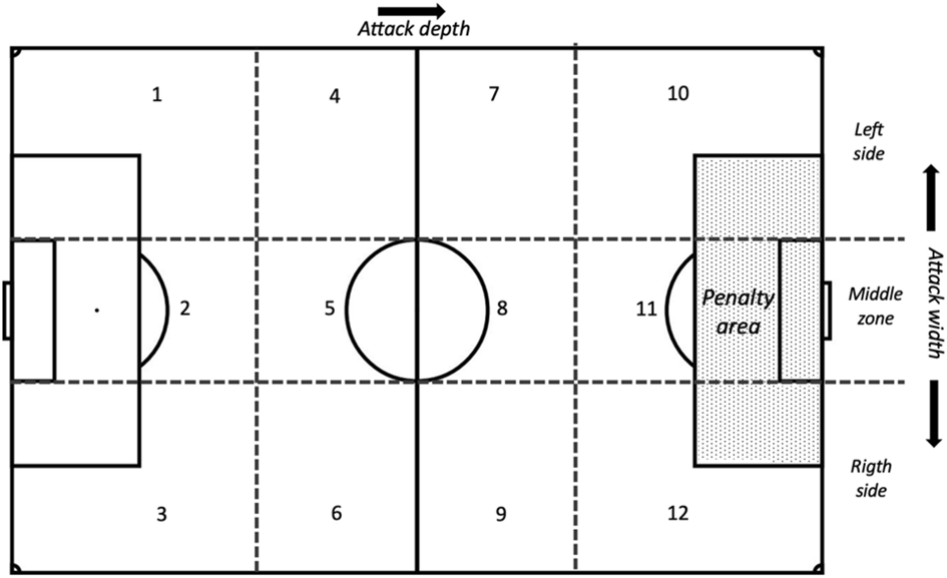
Spatial division of the playing field into launch zones (adapted from Sarmento et al.^23^; Sainz de Baranda et al.^25^).

All matches were recorded using a video camara (Sony, RS100IV) and replayed using Dartfish 10 Live S analysis software (Fribourg, Switzerland). This software enables video recordings to be paused, replayed, played in slow-motion or scrolled frame by frame, thus, tactical individual actions could be observed and tagged accurately^21^. Match observation was undertaken by the study-team’s head coach and one assistant coach (both UEFA-qualified) and data reported using a specifically tailored observational tool and notation system following Sarmento et al.^22^. This tool defines a set of launch zones (see Fig. 2) and player activities are categorized as (a) offensives: shots, passes and dribbles and (b) defensives: interceptions and clearances (see Table 1). As a test of reliability, each coach analysed a test sample of > 100 games. Interobserver reliability was estimated by an independent analyst^23^ who coded 200 randomly selected activities. Intra-observer and interobserver agreement were assessed using Cohen’s Kappa^24^ and demonstrated that the results had a high level of reliability (see Table 2: Kappa values > 0.90).

**Table 1:** Categories and descriptions of variables.

Categories	Variables and definitions
Offensive	Shots: goal shots
	Out of box shots: shots from outside the penalty area
	Goal area shots: shots from inside the goal area
	Short passes: when a player performed a pass within the same zone or one of the contiguous zones to a teammate
	Long passes: when a player performs a pass that crosses 2 contiguous zones and is played in a third zone to a teammate
	Dribbles: attempt by a player to beat an opponent while maintaining possession of the ball
Defensive	Interceptions: when a defensive player blocks a pass from an opponent
	Clearances: when a player kicks the ball away from the goal they are defending

**Table 2:** Kappa values to assess intra-observer and interobserver reliability.

Activities	Intra-observer		Interobserver	
	Kappa	CI (95%)	Kappa	CI (95%)
Shots	0.98	0.96–0.99	0.95	0.93–0.97
Passes	0.98	0.97–0.99	0.97	0.95–0.98
Dribbles	0.96	0.94–0.98	0.90	0.86–0.94
Interceptions	0.93	0.92–0.95	0.87	0.86–0.89
Clearances	0.92	0.91–0.94	0.89	0.88–0.92

### Statistical analysis

Throughout this article, data is presented as a mean ± standard deviation (± SD). Given that matches had different durations depending on the amount of extra time added, to ensure fair comparison between the tactical formations used in each match, variables were normalized for 90 min of game-time. Differences between running performance variables and technical-tactical action variables was assessed using one-way ANOVA by comparing tactical formation. Wilks’ Lambda was calculated and when this indicated a significant F-value, Bonferroni post-hoc test was performed to determine pairwise differences and the Cohen’s *d*^26^ was calculated to determine the magnitude of differences (*ES*) between tactical formation for each variable where: ES < 0.2 represents an insignificant effect size; small and moderate effect sizes are indicated by, respectively *ES* < 0.6 and *ES* < 1.2; large, very large and extremely large effect sizes are indicated by, respectively, *ES* < 2.0, *ES* > 2.0, and *ES* > 4.0^27^. Statistical analyses were performed using SPSS for Windows, Version 25.0 (SPSS Inc., Chicago, IL, USA). The level of statistical significance was set at *p* < 0.05.

## Results

Table 3 show the variables measured (normalized to 90 min game-time) for wingers in all tactical formations. We found statistically significant differences for all variables (*p* < 0.05) except in the cases of TD, Acc > 4 and Out of box shots.

**Table 3:** Descriptive statistics (normalized for 90 min game-time): wingers’ running performance and tactical individual actions dependent upon tactical formation.

Variable	4-2-3-1		4-4-2		3-5-2		3-4-2-1		*F*	*p*
	Mean	SD	Mean	SD	Mean	SD	Mean	SD		
Running performance variables										
TD (km)	10.076	0.870	10.129	0.773	10.168	0.808	9.980	1.017	0.193	0.901
D > 14.4 (m)	2069.686	274.266	2167.732	230.216	2036.160	118.375	1962.008	316.413	3.211	0.026*
D > 19.8 (m)	670.300	61.231	729.510	73.203	741.090	43.099	605.622	43.899	25.147	0.000*
D > 25 (m)	124.258	20.938	143.669	46.508	153.926	14.363	124.996	22.699	5.495	0.001*
Acc2-4 (n)	140.663	22.007	160.967	25.644	166.270	13.526	153.658	58.637	3.422	0.020*
Acc > 4 (n)	28.562	6.065	28.311	6.581	24.783	4.525	29.958	10.647	1.868	0.139
Tactical individual actions										
Shots (n)	3.136	2.732	2.970	2.671	1.103	1.032	3.924	3.594	3.751	0.013*
Out of box shots (n)	1.346	1.803	1.107	1.091	0.829	0.767	1.480	1.910	0.772	0.512
Goal area shots (n)	1.790	2.202	1.863	2.483	0.274	0.458	2.444	2.943	3.168	0.027*
Short passes (n)	26.000	6.676	32.047	9.628	32.277	13.590	36.580	13.756	5.180	0.002*
Long passes (n)	2.658	2.932	4.474	4.491	3.628	0.900	2.519	2.201	2.547	0.060
Dribbles (n)	4.399	2.315	3.199	2.881	2.116	1.433	5.656	5.131	5.138	0.002*
Interceptions (n)	6.665	3.203	8.879	5.553	8.909	5.470	3.044	1.946	9.685	0.000*
Clearances (n)	1.364	1.741	3.039	3.790	2.452	1.272	2.328	1.913	2.700	0.049*

*Min.* minutes, *TD* the total distance, *D > 14.4* the distance covered between 14.4 and 19.8 km/h, *D > 19.8* the distance covered between 19.9 and 25 km/h, *D > 25* the distance covered above 25 km/h, *Acc2-4* number of accelerations between 2–4 ms-2, *Acc > 4* number of accelerations above 4 ms-2, *F* F-value, *p* p-value.

*p < 0.05.

**Figure 3:**
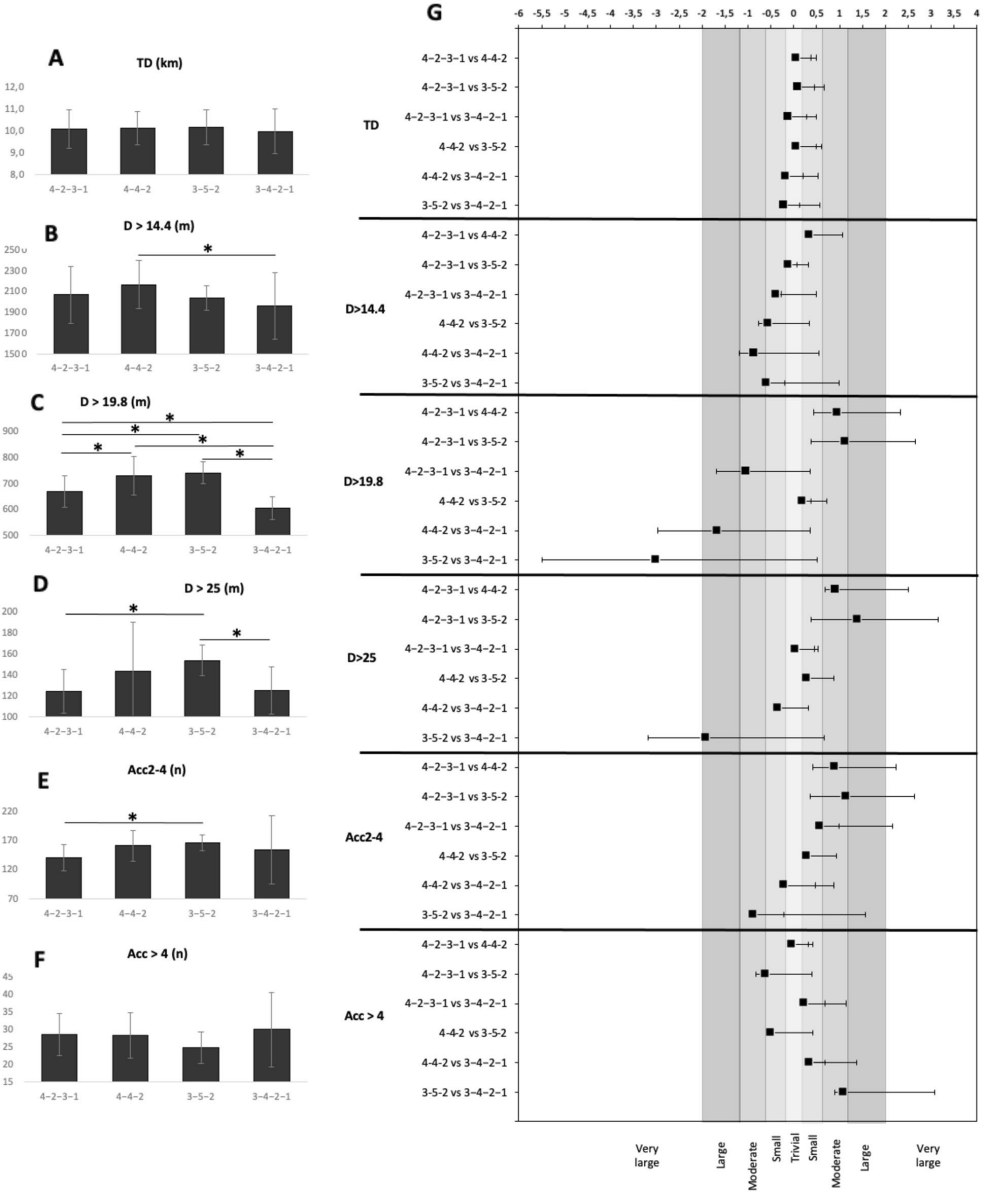
Differences between running performance variables depending on tactical formation: Data normalized to 90 min game-time (panels (**A–F**)) and effect sizes (panel (**G**)). The different tones of grey represent different effect sizes (light to dark: smallest to largest effect size) and the unshaded area represents the most significant effect size. *p < 0.05.* Min.* minutes,* TD* the total distance,* D > 14.4* the distance covered between 14.4 and 19.8 km/h,* D > 19.8* the distance covered between 19.9 and 25 km/h,* D > 25* the distance covered above 25 km/h,* Acc2-4* number of accelerations between 2–4 ms^−2^,* Acc > 4* number of accelerations above 4 ms^−2^.

Results of the Bonferroni post-hoc test and the magnitude of the differences between running performance with respect to tactical formation are shown in Fig. 3. The Bonferroni post-hoc test identified differences for D > 14.1 comparing 4-4-2 and 3-4-2-1 (*p* = 0.019); for D > 19.8 comparing 4-2-3-1 and 4-4-2 (*p* = 0.000), 4-2-3-1 and 3-5-2 (*p* = 0.000), 4-2-3-1 and 3-4-2-1 (*p* = 0.000), 4-4-2 and 3-4-2-1 (*p* = 0.000), and 3-5-2 and 3-4-2-1 (*p* = 0.000); for D > 25.0 comparing 4-2-3-1 and 3-5-2 (*p* = 0.006), and 3-5-2 and 3-4-2-1 (*p* = 0.021); and, finally, for Acc2-4 comparing 4-2-3-1 and 3-5-2 (*p* = 0.044).

Effect sizes were all insignificant or small for TD, while the effect sizes for D > 14.4, Acc2-4 and Acc > 4 reached moderate values. However, for D > 19.8, large effect sizes were seen comparing 4-4-2 and 3-4-2-1 (*ES* = − 1.66) with very large effect sizes recorded for the comparison of 3-5-2 and 3-4-2-1 (*ES* = − 3.00). In addition, for D > 25 large effect sizes were seen comparing 4-2-3-1 and 3-5-2 (*ES* = 1.39), and 3-5-2 and 3-4-2-1 (*ES* = − 1.92).

**Figure 4:**
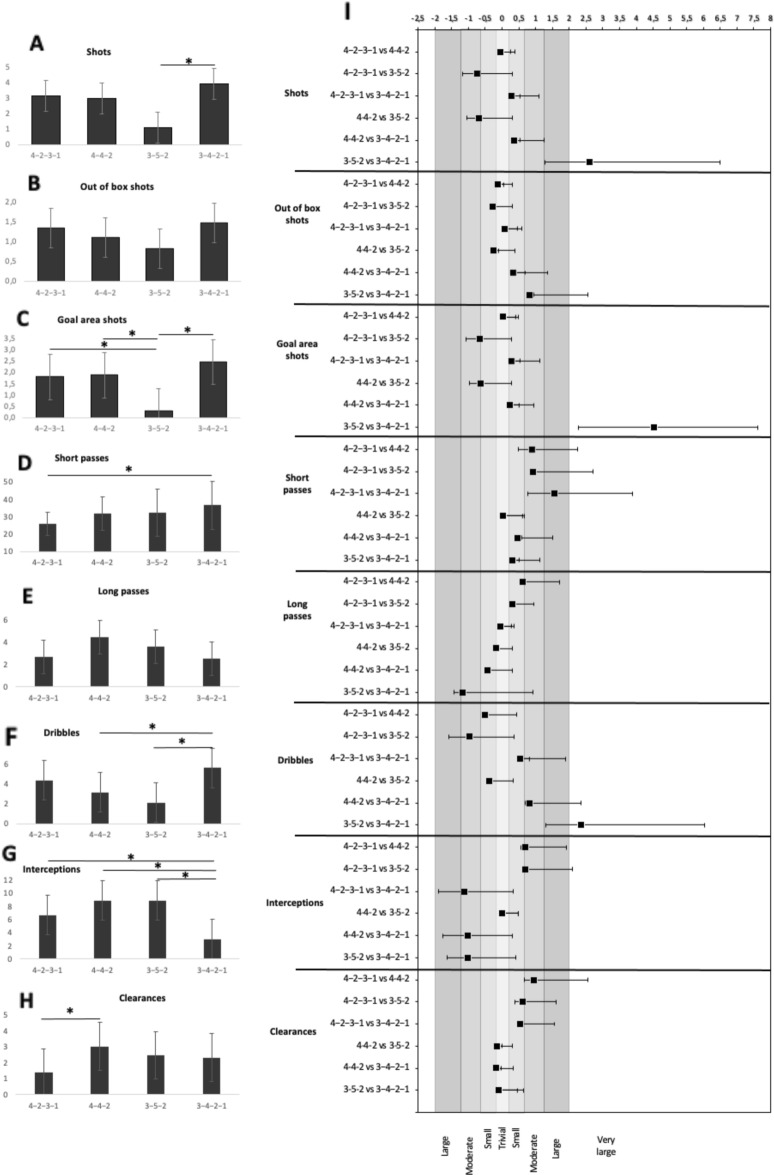
Differences between technical-tactical action variables depending on tactical formation: Data normalized for 90 min game-time (panels (**A–H**)) and effect size values (panel (**I**)). The different tones of grey represent different effect sizes (light to dark: smallest to largest effect size) and the unshaded area represents the most significant effect size. *p < 0.05.

Concerning differences in tactical individual actions with respect to the various tactical formations used, Bonferroni post-hoc tests and effect size calculations (Fig. 4) identified the following statistically significant differences: for Shots comparing 3-5-2 and 3-4-2-1 (*p* = 0.008; *ES* = 2.61); for Goal Area Shots comparing 3-5-2 and 3-4-2-1 (*p* = 0.021; *ES* = 4.52); for Short Passes comparing 4-2-3-1 and 3-4-2-1 (*p* = 0.001; *ES* = 1.55); for Dribbles comparing 4-4-2 and 3-4-2-1 (*p* = 0.029; *ES* = 0.83) and comparing 3-5-2 and 3-4-2-1 (*p* = 0.003; *ES* = 2.36); for Interceptions comparing 3-4-2-1 with 4-2-3-1 (*p* = 0.012; *ES* = − 1.11), 4-4-2 (*p* = 0.000; *ES* = − 1.03) and 3-5-2 (*p* = 0.000; *ES* = 0.94); and finally, for Clearances comparing 4-2-3-1 and 4-4-2 (*p* = 0.035; *ES* = 0.94).

## Discussion

This study followed an amateur male football team over a complete season with the purpose of examining the differences in running performance demands and the performance of tactical individual actions by wingers depending on the team’s tactical formation. The main result to highlight is that, compared to every other tactical formation, 3-5-2 results in higher running demands across all variables, with the exception of High accelerations. Differences are statistically significant in several instances, in particular, comparing this formation to 3-4-2-1, where, in addition, very large ES are observed for the parameters Running and Sprinting. Furthermore, comparing 3-5-2 and 4-2-3-1, a moderate effect size is seen for Medium accelerations. With regards to High accelerations, it is interesting to note that 3-5-2 actually results in the lowest score for this variable (its highest value is recorded for 3-4-2-1). In terms of tactical individual actions, formations 3-5-2 and 3-4-2-1 wingers register the lowest and the highest values, respectively, in those variables directly related to goal scoring: Out of Box Shots and Goal Area Shots, and Dribbles. Finally, for Interceptions, here the playing formation 3-4-2-1 shows much lower values compared to other tactical formations. These findings reported turn out to be consistent with the hypothesis that wingers in 3-5-2 require the highest physical demands, and in 3-4-2-1 perform high actions related to the goal.

The variations in running performance detected are due to the specific demands of each playing formation. It is perhaps surprising then that no statistical differences between formations were observed for TD. The lack of variation in TD between tactical formations is, however, consistent with the results of Bradley et al.^28^, who examined three tactical formations (4-3-3, 4-4-2 and 4-5-1) and demonstrates no statistical differences between them in terms of total distances run. In contrast, in a study of Italian elite wingers, Riboli et al.^29^ showed that the formation 3-5-2 demanded higher TDs than either 3-4-1-2 or 4-4-2.

Regarding physical performance indicators, Bradley & Ade^30^ propose that more attention should be paid to the tactical activities’ players perform. Our data concerning high intensity running tends to bear this out. Specifically, this study demonstrates that the tactical formation 3–5-2 exerts the highest performance demands in terms of high running intensity, particularly for D > 19.8 (*F* = 25.147; *p* = 0.000), D > 25.0 (*F* = 5.495; *p* = 0.001) and Acc2-4 (*F* = 3.422; *p* = 0.020). Football wingers in this playing formation support attacking forwards, but, in comparison to other formations, 3–5-2 places wingers several steps behind their forwards. In this way, high intensity sprints are required to make up for the fact that they must start so far behind and thus help wingers to break the defensive line while keeping pace with the forwards. In addition, wingers in 3–5-2- also need to support more often defensive actions dropping to the last line to provide a 5 defensive line, and therefore, including more high-intensity running to cover backspace. The next most demanding formation in terms of running appears to be that of 4–4-2. In this formation, wingers are located in closed positions in order to help in the defensive phase so reducing the number of high intensity running activities that might be required^9^.

The lowest values for high intensity running are obtained for formation 3-4-2-1. Our findings are somewhat supported by those of work by Riboli et al.^29^ that looked at elite winger performance in various tactical formations and observed that 3-4-2-1 resulted in the second lowest distances run at high intensity (> 24 kmh^−1^) with the formation 4-3-3 being the only one that produced lower values. Interestingly, compared to all other formations, 3-4-2-1 shows the highest values for Acc > 4. We would suggest that this might be explained by the particular characteristics of this formation, specifically, the presence of only one forward at the front with the two wingers in close proximity on either side. This means that wingers can focus on short distance, multidirectional movements—involving explosive acceleration—in the goal scoring area^9^. Also, we could suggest that the movements of wingers in 3-4-2-1 formation depend on the midfielders who specifically play on the sides.

Regarding tactical individual actions, here wingers perform most offensive activities—Goal area shots, Short passes, and Dribbles—in the 3-4-2-1 formation and most Long passes in 4-4-2. The lowest value of Goal area shots is found for formation 3-5-2 and this may be explained by the importance of wingers’ defensive role in this formation. This particular tactical formation gives the team only two wide players who have to cover the whole flank, whereas in the other playing formations analyzed, this task is divided among a total of four players (two on each side)^31^. Wingers perform the highest number of Short passes in the 3-4-2-1 formation and the difference is statistically significant for the comparison with 4-2-3-1 (*p* = 0.001; *ES* = 1.55). Findings in a study by Arjol-Serrano et al.^9^ also show that the 4-2-3-1 formation results in few Short passes, however their work considers midfielders rather than wingers and the comparison is with the 4-4-2 formation. With the team in a 4-4-2 formation, it appears that wingers perform the largest number of Long passes but produce low numbers of Dribbles, although the lowest value for this variable was for 3-5-2. In this formation, wingers are not always located in the flanks and thus it is likely they engage in more offensive actions in the goal-creation area keeping possession of the ball and moving forward^9^.

Concerning defensive activities, wingers perform best in Interceptions (*F* = 9.685; *p* = 0.000) and Clearances (*F* = 2.700; *p* = 0.049) for the 4-4-2 and 3-5-2 formations. This can be explained as, on one hand, in the diamond-shaped 4-4-2 formation wingers are located principally in closed positions which assists defensive activities^9^ while on the other hand, the 3-5-2 formation allows wingers to travel deep into their own half of the pitch and create a five-player strong defensive line with the three central defenders from which defensive actions can be progressed. The lowest number of Interceptions is recorded for the 3-4-2-1 formation with statistically significant differences compared to other formations. This formation which uses only 3 defensive players, requires additional players to become involved in defense and, in this case, that task is assumed by the line of 4 midfielders^32^. Consequently, wingers in this formation are freed up to contribute to the attack, however, their greater participation in offensive actions is potentially to the detriment of defensive activities.

Our study has two main limitations. First, we were unable to evaluate other common playing formations used in football (e.g. 4-1-4-1). Secondly, we did not consider the opposing teams’ skill level and the influence of their tactical formation on the study-team’s performance. Therefore, future studies in order to examine further the physical and tactical responses of amateur football players, including other playing positions, the level of the teams (our own and opponent), or female sample. Despite these shortcomings, our study has significant value as the first study to analyze such a comprehensive range of performance indicators and tactical individual actions and to evaluate them with respect to playing formation for a particular playing position. The study is also novel in its consideration of a complete season in an amateur context.

## Conclusion

The results of the current research show evidence that playing formation influences the physical demands and tactical individual actions of wingers playing on an amateur football team. Key results include the finding that the 3-5-2 formation requires the highest running demands and while 3-4-2-1 produces the more offensive activities than other formations, specifically Shots, Short passes, and Dribbles (although not Long passes), 4-4-2 results in the highest numbers of defensive actions (Interceptions and Clearances). Understanding the particular physical demands and technical-tactical activities associated with or promoted by different formations makes the present findings of potential practical use to coaches in assessing match demands. This work focusses on wingers, and we feel that adopting a position-specific approach to player conditioning more generally could benefit teams. Performance analysis for a given set of players as completed for this work concentrating on wingers might be used (as a practical application) to help coaches tailor their training methods to the needs of those specific players and this in turn could enhance the tactical performance of the team overall. For instance, for coaches working on 3-5-2 formation, this position-specific training should occur for wingers to develop high intensity actions on the sides to the field; on 3-4-2-1, training exercises should focus on developing skills that are relevant to the gold zone, such as executing dribbles, short passes or taking shots on goal, all of them under an opponent’s pressing. Finally, on 4-4-2, the training tasks should enhance the defensive skills of the wingers.

## Data Availability

The data analysed during the current study are available on link: https://shorturl.at/mnXY2.
